# Diversifying molecular and topological space via a supramolecular solid-state synthesis: a purely organic mok net sustained by hydrogen bonds

**DOI:** 10.1107/S2052252519011382

**Published:** 2019-09-07

**Authors:** Shalisa M. Oburn, Michael A. Sinnwell, Devin P. Ericson, Eric W. Reinheimer, Davide M. Proserpio, Ryan H. Groeneman, Leonard MacGillivray

**Affiliations:** a Department of Chemistry, University of Iowa, Iowa City, IA 52242, USA; b Department of Biological Sciences, Webster University, St. Louis, MO 63119, USA; c Department of Chemistry and the W. M. Keck Foundation Center for Molecular Structure, California State University, San Marcos, CA 92096, USA; d Dipartimento di Chimica, Università degli Studi di Milano, Milano 20133, Italy; e Samara Center for Theoretical Materials Science (SCTMS), Samara State Technical University, Samara 443100, Russia

**Keywords:** supramolecular chemistry, [2+2] photo­cyclo­addition, three-dimensional hydrogen-bonded organic networks, crystal engineering, intermolecular interactions, co-crystals, organic solid-state reactions

## Abstract

A three-dimensional hydrogen-bonded network based on a rare **mok** topology has been constructed using an organic molecule synthesized in the solid state. The molecule is obtained using a supramolecular protecting-group strategy that is applied to a solid-state [2+2] photodimerization. The photodimerization affords a novel head-to-head cyclo­butane product. The cyclo­butane possesses tetrahedrally disposed *cis*-hydrogen-bond donor (phenolic) and *cis*-hydrogen-bond acceptor (pyridyl) groups. The product self-assembles in the solid state to form a **mok** network that exhibits twofold interpenetration. The cyclo­butane adopts different conformations to provide combinations of hydrogen-bond donor and acceptor sites to conform to the structural requirements of the **mok** net.

## Abstract 2

A hydrogen-bond-directed [2+2] photodimerization performed in the solid state is used to generate a head-to-head (HH) cyclo­butane photoproduct functionalized with *cis*-phenolic and *cis*-pyridyl groups. The photoproduct self-assembles as a pure form in the solid state to form a rare three-dimensional hydrogen-bonded network with a topology that conforms to a **mok** net. The construction of the HH cyclo­butane is achieved using a newly introduced supramolecular protecting-group strategy.

## 1. Introduction   

Efforts of chemists to develop new avenues to form covalent bonds and generate molecules that diversify chemical space are increasingly important (*e.g.* materials science, medicine; Dobson, 2004 ▸). In this context, chemical reactions performed in organic crystals can be used to synthesize molecules that possess functional groups with stereochemical relationships which are not accessible in solution (Elacqua *et al.*, 2012 ▸; Oburn *et al.*, 2017 ▸). The stereochemical outcome of a reaction in the crystalline state is generally dictated by topological arrangement or supramolecular organization in a lattice (Biradha & Santra, 2013 ▸; Ramamurthy & Sivaguru, 2016 ▸; Vittal & Quah, 2017 ▸). A well established reaction to proceed in the solid state is the [2+2] photodimerization of alkenes that generates carbon–carbon single (C—C) bonds in the form of cyclo­butane rings. The reaction is a mainstay for crystal engineers that seek to form covalent bonds in solids. In recent years, cyclo­addition, when controlled by hydrogen bonds (*e.g.* resorcinol or res templates) and principles of self-assembly, has enabled the synthesis of complex organic molecules based on unique topologies (*e.g*. ladderanes; Sinnwell *et al.*, 2015 ▸; Lange *et al.*, 2017 ▸; Gao *et al.*, 2004 ▸).

**Figure 1:**
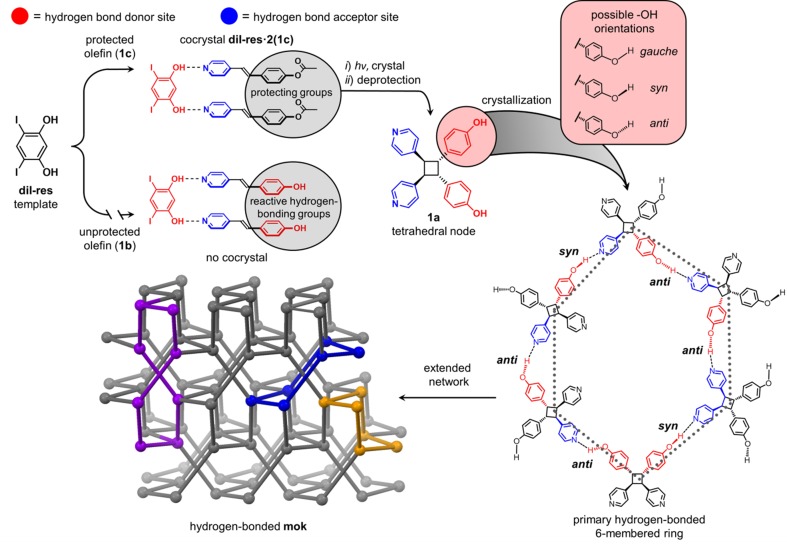
Post-installation of phenolic groups.

A current goal of crystal engineers, and general efforts of solid-state chemists, is to design molecular building blocks that self-assemble to form extended (*i.e.* one-, two- and three-dimensional) network topologies. Of particular interest are entangled nets, wherein nodes are linked to form intertwined and self-catenated structures. The **mok** net, which is comprised of tetrahedral nodes linked in three-dimensions – akin to the well known diamondoid net – is one such example (Fig. 1 ▸) (O’Keeffe, 1991 ▸; Alexandrov *et al.*, 2012 ▸; Bonneau & O’Keeffe, 2015 ▸). Self-catenation of the **mok** net is based on the shortest rings (six-membered rings, blue), with interpenetration of two hexagonal (**hcb**) subnets of the **mok** net effectively facilitating the self-catenation. A consequence of the self-catenation is the generation of two additional rings (six- and eight-membered, orange and purple, respectively). Importantly, the **mok** net has only been observed in assembly processes based on metal–organic components wherein the tetrahedral node is supplied by a metal center (Gong *et al.*, 2011 ▸; Zhang *et al.*, 2015 ▸; Liang *et al.*, 2013 ▸). Moreover, the mok net has been recognized by O’Keeffe as ‘likely to be difficult to achieve chemically’ in the solid state due to its intricacy. An organic molecule that fulfills the role of the tetrahedral nodes in a **mok** net has not yet been identified.

With this in mind, we report here the supramolecular solid-state construction of the organic molecule rctt-1,2-bis­(4-pyridyl)-3,4-bis­(4-phenol)cyclo­butane (**1a**), showing it self-assembles in the solid state to form a network that conforms to the **mok** topology. The synthesis is achieved using a novel supramolecular protecting-group strategy applied to phenols and utilizes 4,6-di­iodo-res (diI-res) as a hydrogen-bond donor template (Elacqua *et al.*, 2012 ▸). This strategy enables protection of terminal phenolic groups from participating in hydrogen bonds in the solid state and then post-installation of *cis*-phenolic groups onto a cyclo­butane ring system (Fig. 1 ▸). We show that the head-to-head (HH) cyclo­butane **1a**, following removal from the molecular template, self-assembles as a pure form to produce a hydrogen-bonded twofold interpenetrated net of **mok** topology. Within the network, the cyclo­butane ring of **1a** acts as a node with the radial phenolic and pyridyl groups serving as hydrogen-bond donor and acceptor linkers, respectively, resulting in the first purely organic **mok** network (Gong *et al.*, 2011 ▸; Liang *et al.*, 2013 ▸; Zhang *et al.*, 2015 ▸; Li *et al.*, 2017 ▸).

## 2. Results and discussion   

The HH cyclo­butane **1a** contains *cis*-4-phenolic and *cis*-4-pyridyl groups. Although cyclo­butanes functionalized with *cis*-4-pyridyl groups have been synthesized in the solid state using hydrogen bonds with res templates, the template-directed synthesis of a cyclo­butane lined with phenolic groups has not yet been reported. We note that the synthesis of **1a** itself has not been reported in either solution or the solid state, although a photodimerization of protonated 1b (1b = *trans*-1-(4-phenol)-2-(4-pyridyl)­ethyl­ene) in HCl has been shown to yield a mixture of head-to-tail (HT) isomers (Zhang *et al.*, 2000 ▸). To us, **1a** was attractive as a building block in supramolecular chemistry given the presence of the radial and tetrahedrally disposed hydrogen-bond donor (phenol) and acceptor (pyridyl) groups. We expected the groups to equip **1a** with a capacity to form 4-connected nets (*e.g.* diamondoid) (Ermer, 1988 ▸; Baburin *et al.*, 2008 ▸). Many conformations furnished by the hydrogen-bond donor groups would equip **1a** with a capacity to form different 4-connected nets. While 1b has been a subject of numerous studies (*e.g.* liquid crystals), we were also surprised that the crystal structure of 1b had not been reported.

The ability of the symmetrical cyclo­butane rctt-tetra­kis­(4-pyridyl)­cyclo­butane (tpcb) (*D*_2*h*_ symmetry) to serve as a tetrahedral node of extended nets composed of metal and organic building blocks was originally elucidated by Schroder and Champness (Blake *et al.*, 1997 ▸). Specifically, tpcb served as a 4-connected node to support a net of composition [Ag(tpcb)]BF_4_ (Blake *et al.*, 1997 ▸; Liu *et al.*, 2011 ▸). We expected the cyclo­butane **1a**, being of lower symmetry (*C_s_*), to be able to interact with itself, in contrast to tpcb, by way of complementary hydrogen-bond donor and acceptor groups. The presence of the donor and acceptor sites attached to the cyclo­butane ring would equip the molecule with a capacity to self-assemble into a net purely organic in composition.

### 2.1. Photostable parent alkene 1b   

**Figure 2:**
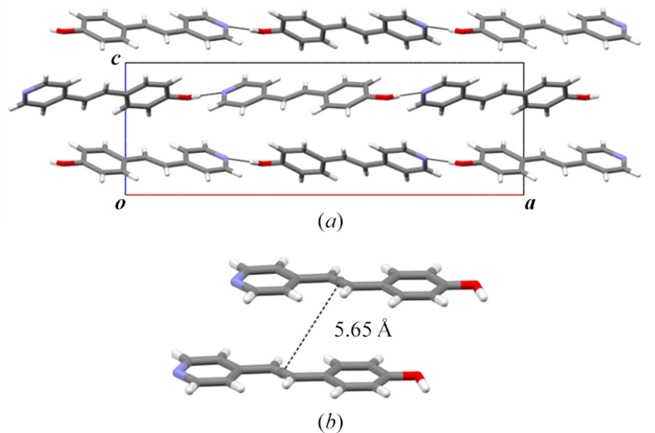
X-ray structure of photostable 1b: (*a*) hydrogen-bonded chains and (*b*) stacked C=C bonds of nearest-neighbour alkenes.

Plate-like single crystals of 1b were grown by slow evaporation in MeOH/ethyl acetate (1:1, *v*:*v*) over a period of 10 d, crystallizing in the orthorhombic space group *P* *ca*2_1_. The molecule adopts a planar conformation (twist: 1.63°) with the hydroxyl and pyridyl groups participating in intermolecular O—H⋯N hydrogen bonds [O⋯N, O—H⋯N: 2.729 (5) Å, 177.8 (2)°] (Fig. 2 ▸). The alkene self-assembles to form chains along the *a* axis that stack HH and edge-to-face. Nearest-neighbor C=C bonds are separated by 5.65 Å, which is beyond the limit of the work by Schmidt (1971 ▸). When subjected to UV-radiation (450 W medium-pressure Hg lamp) for a period of up to 50 h, 1b was determined to be photostable.

### 2.2. Attempts to form cocrystals of 1b   

While 1b is photostable, attempts to cocrystallize 1b with diI-res and, in doing so, form a cocrystal with 1b stacked HH, to react to form the cyclo­butane **1a** were unsuccessful (Fig. 1 ▸). Liquid-assisted grinding of 1b with diI-res afforded a mixture of the two solids, as demonstrated by powder X-ray diffraction. Attempts to grow cocrystals from solution were also unsuccessful. The solution crystallization experiments typically produced a powder that was identified as the alkene 1b. We attributed the inability of diI-res to form a cocrystal with 1b to the inability of diI-res to compete with the hydrogen bonding between the phenolic and pyridyl groups present in crystalline 1b [Fig. 2 ▸(*a*)] (Elacqua *et al.*, 2012 ▸).

### 2.3. Supramolecular protecting-group strategy   

While 1b is photostable as a pure solid, we determined that the C=C bonds of 1b are made photoactive when the protected methyl ester **1c** (**1c** = *trans*-1-(4-acet­oxy)-2-(4-pyridyl)­ethyl­ene) is cocrystallized with diI-res in a newly designed supramolecular protecting-group strategy (Elacqua *et al.*, 2012 ▸). For the strategy, we aimed to develop a method that would allow us to mask the hydrogen bonding ability of the OH group and, at the same time, have minimum steric impact on the requirement of the C=C bonds to stack parallel and on the order of 4.2 Å. Given that cinnamates are known to stack and photodimerize in the solid state (Lewis *et al.*, 1984 ▸), we targeted the ester linkage. Specifically, we expected acyl­ation of the phenol moiety of 1b to allow the C=C bonds of 1b in the form of **1c** to stack in the solid state and conform to the topochemical postulate for a photoreaction. Acyl­ation of 1b was thus performed and afforded **1c** in high yield (Yin *et al.*, 2011 ▸).

### 2.4. Photostable protected alkene 1c   

**Figure 3:**
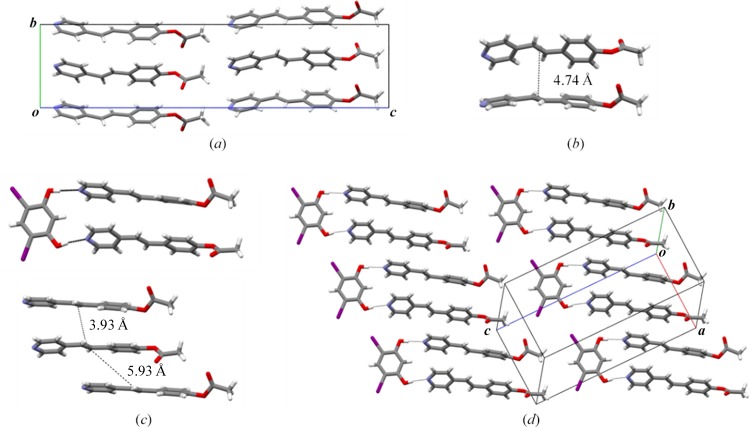
X-ray structures of **1c** and (diI-res)·2(**1c**): (*a*) edge-to-face forces of **1c**, (*b*) C=C bond interactions of nearest-neighbour alkenes of **1c**, (*c*) hydrogen-bonded three-component assembly (diI-res)·2(**1c**) (top) with C=C separations (bottom) and (*d*) two-dimensional sheets of (diI-res)·2(**1c**).

Plate-like single crystals of **1c** were generated by slow evaporation in ethyl acetate/ethanol (3:2, v:v) over a period of 2 d. As in the case of 1b, the alkene **1c** is photostable [Fig. 3 ▸(*a*)] and crystallizes in the orthorhombic space group *Pbca*. The aromatic rings lie approximately coplanar (twist: 4.66°) with the acet­oxy group twisted from coplanarity (twist: 63.9°). The alkene packs HH and edge-to-face with the nearest C=C bonds separated by 4.74 Å, which is also beyond the limit of the work by Schmidt (1971 ▸) [Fig. 3 ▸(*b*)]. UV-radiation for up to 50 h revealed **1c** to be photostable.

### 2.5. Photoreactive cocrystal **1c**   

Although **1c** as a pure form is photostable, cocrystals of the alkene using the supramolecular protecting-group strategy with diI-res are photoactive and generate the cyclo­butane 1d [where: 1d = 1,2-bis­(4-pyridyl)-3,4-bis­(4-acet­oxy­phenyl) cyclo­butane] regioselectively and in quantitative yield.

Single crystals of (diI-res)·2(**1c**) in the form of colorless plates were formed by combining solutions of **1c** (50 mg, 0.21 mmol) in ethyl acetate (3 ml) and diI-res (56 mg, 0.16 mmol) in EtOH (2 ml). The components of (diI-res)·2(**1c**) crystallize in the triclinic space group 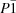. The molecules form three-component assemblies sustained by two O—H⋯N hydrogen bonds [O⋯N, O—H⋯N: 2.677 (5) Å, 167.1 (4)°; 2.725 (5) Å, 172.6 (3)°] [Fig. 3 ▸(*c*)]. The rings of the alkene, in contrast to 1b and pure **1c**, stack HH and face-to-face with the acet­oxy groups twisted from planarity (twists: 41.8, 74.1°). The stacked C=C bonds lie parallel and are separated by 3.93 Å, which conforms to the geometry determined by Schmidt (1971 ▸). The assemblies interact via a halogen bond (Metrangolo & Resnati, 2014 ▸) [I⋯O: 3.309 (3), 3.227 (4) Å, θ = 159.1 (1), 174.8 (1)°] to give two-dimensional sheets in the crystallographic *ac* plane with neighboring C=C bonds separated by 5.93 Å.

To determine the reactivity of (diI-res)·2(**1c**), a finely ground crystalline powder was spread between two glass plates and exposed to broadband UV irradiation. A ^1^H NMR spectrum revealed the complete disappearance of alkene signals (7.19 and 7.56 p.p.m.) and the appearance of a cyclo­butane signal (4.58 p.p.m.) following 100 h of UV-irradiation (see supporting information).

**Figure 4:**
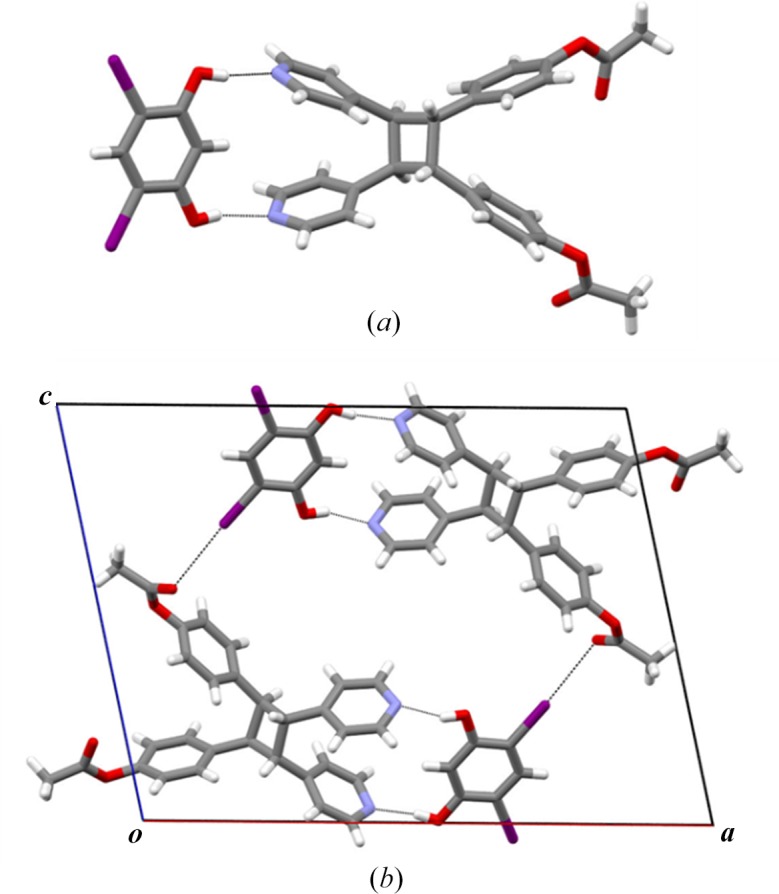
X-ray structure of (diI-res)·(**1d**): (*a*) hydrogen bonds and (*b*) I⋯O halogen bonds.

To determine the stereochemistry of the photoproduct, single crystals as colorless prisms were obtained by recrystallization of the reacted solid from ethanol/ethyl acetate (1:1, v:v) over a period of 3 d. The components of (diI-res)·(1d) crystallize in the monoclinic space group P2_1_/*c* with the stereochemistry being confirmed as the *rctt* isomer 1d (Fig. 4 ▸). The solid is composed of two-component assemblies sustained by two O—H⋯N hydrogen-bonds [O⋯N, O—H⋯N: 2.658 (6) Å, 153.4 (3)°; 2.704 (6) Å, 170.9 (3)°], with I⋯O halogen bonds also formed involving the carboxyl O atom of 1d [I⋯O: 3.442 (9) Å, θ = 144.9 (2)°]. Additionally, I⋯O halogen bonds are present involving a hydroxyl O atom [I⋯O: 3.436 (4) Å, θ = 152.6 (2)°] to generate ribbons along the crystallographic *c* axis.

### 2.6. Rare organic **mok** net   

The synthesis of the targeted unsymmetrical cyclo­butane **1a** was next achieved in the deprotection of 1d by treating the photoreacted solid of (diI-res)·(1d) with NaOH as base (see supporting information). Single crystals of **1a** suitable for single-crystal X-ray diffraction were obtained by slow solvent evaporation from solution of aqueous MeOH over a period of 5 d.

**Figure 5:**
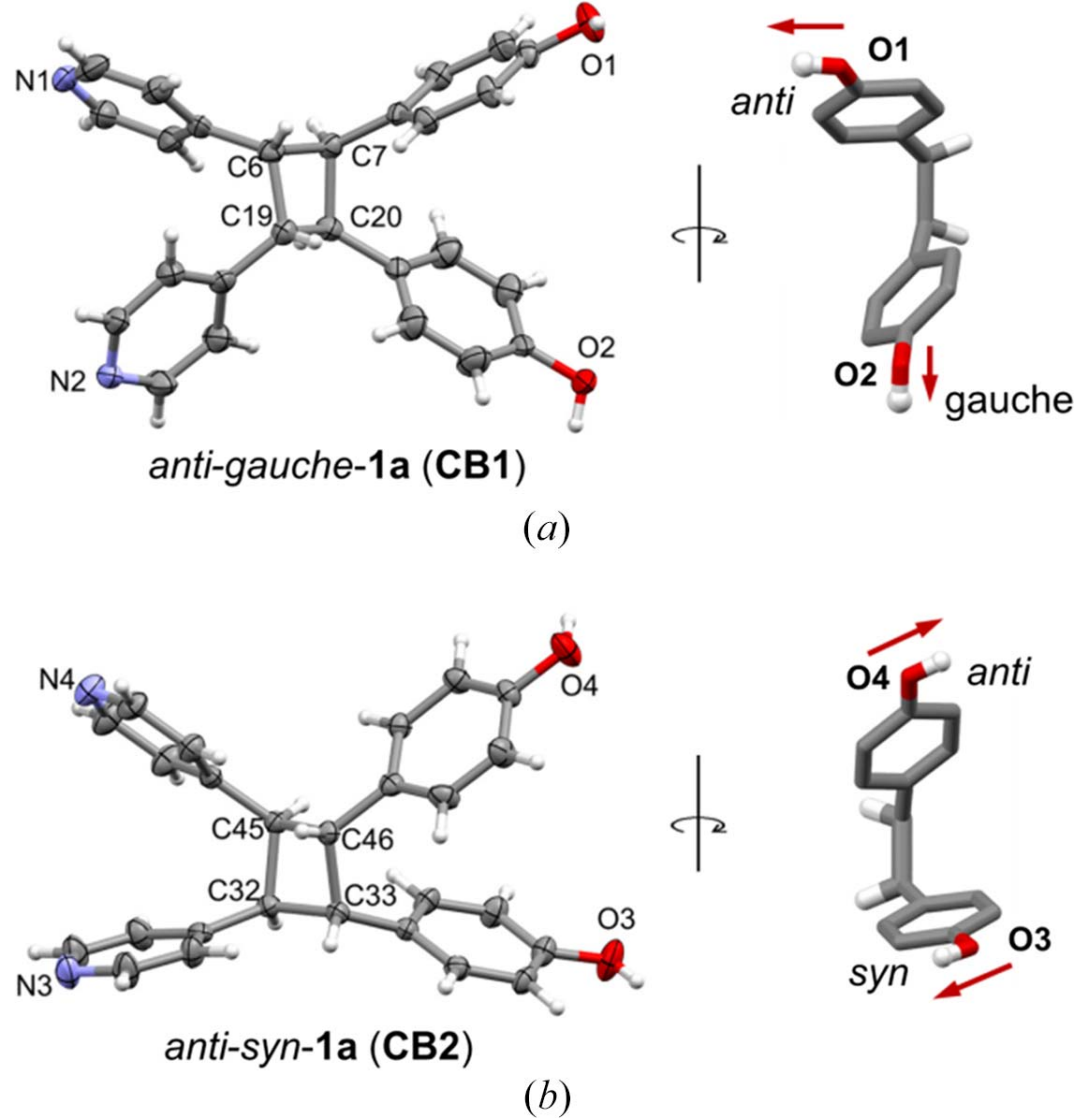
X-ray structure of **1a**: (*a*) *anti–gauche* **1a** (**CB1**) and (*b*) *syn–anti* **1a** (**CB2**). Note: *anti* and *syn* are designated relative to the pyridyl groups.

**Figure 6:**
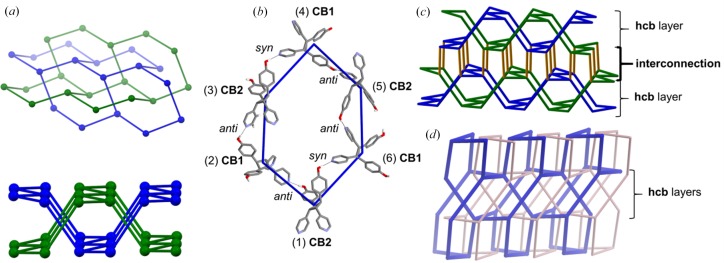
X-ray structure of **mok** topology of **1a**: (*a*) interpenetration of hexagonal (**hcb**) sub-nets highlighted in green and blue, (*b*) building blocks of cyclo­butanes as nodes to form hydrogen-bonded hexagons numbered in a clockwise manner (hydrogens removed for clarity), (*c*) connections of two **hcb** nets (connection highlighted in orange and **hcb** nets in blue/green), and (*d*) twofold interpenetrated **mok** nets highlighted separately in tan and blue.

The asymmetric unit of **1a** consists of two unique cyclo­butanes (**CB1** and **CB2**) that crystallize in the monoclinic space group *I*2/*a*. The deprotection of 1d with the removal of the acet­oxy protecting group confirmed the *rctt* stereochemistry of the cyclo­butane ring of **1a** (Fig. 5 ▸). A remarkable feature of the crystal structure of **1a** is that the cyclo­butane self-assembles to generate a three-dimensioanl hydrogen-bonded framework of **mok** topology (point symbol 6^5.8^). The nodes of the **mok** are defined by the centroids of the cyclo­butane rings of **1a** (Fig. 6 ▸) (Blatov *et al.*, 2014 ▸). The cyclo­butanes provide tetrahedrally disposed and *cisoid* hydrogen-bond donor and acceptor sites to form the 4-connected net.

The pattern of hydrogen bonding that defines the **mok** net of **1a** is complex. The complexity arises since the hydroxyl groups of **1a** adopt two different conformations – **CB1** and **CB2** – within the net. Each conformation is based on the relative dispositions of the hydroxyl groups of each molecule (Fig. 5 ▸). More specifically, **CB1** adopts an *anti*–*gauche* conformation wherein the hydroxyl groups are *anti* and *gauche* relative to the *cis*-4-pyridyl groups [Fig. 5 ▸(*a*)]. The phenyl group related to the *gauche* orientation of **CB1** is disordered [occupancies: site A 0.90 (1); site B 0.10 (1); see supporting information]. **CB2** adopts an *anti*–*syn* conformation whereby the hydroxyl groups are *anti* and *syn* relative to the *cis*-4-pyridyls [Fig. 5 ▸(*b*)]. The cyclo­butane self-assembles to form the **mok** network with all OH and *N*-pyridyl groups participating in O—H⋯N hydrogen bonds (Table 1 ▸).

The self-catenation of the **mok** net arises from interconnection of two twofold interpenetrated **hcb** layers [blue and green, Fig. 6 ▸(*a*)]. The self-assembly of the cyclo­butanes is manifested with **CB1** and **CB2** alternating as adjacent nodes throughout the net [Fig. 6 ▸(*b*)]. All rings of the **mok** network are thus composed of **CB1** and **CB2** which alternate via the O—H⋯N hydrogen bonds.

**Figure 7:**
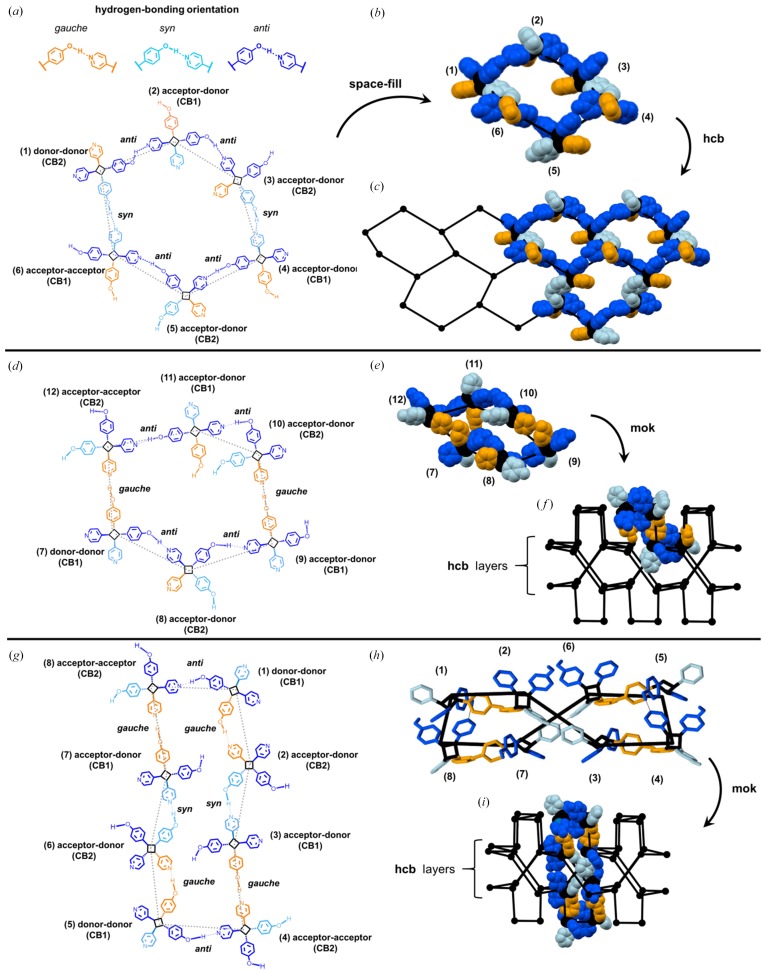
Hydrogen bonding and rings of **mok** net **1a**: (*a*) three linkages with **CB1** and **CB2** (light blue = *syn* linkage; dark blue = *anti* linkage; orange = *gauche* linkage) of a primary six-membered ring, (*b*) space-filling of primary six-membered ring, (*c*) primary six-membered ring showing linkages within **hcb** subnet, (*d*) two types of linkages of a secondary six-membered ring, (*e*) space-filling view of secondary six-membered ring, (*f*) highlighted secondary six-membered ring within **mok** net, (*g*) three types of linkages within an eight-membered ring, (*h*) stick-view of eight-membered ring with *anti*-orientation from **CB1**, and (*i*) space-filling of eight-membered ring showing two interdigitated **hcb** subnets (hydrogen atoms omitted for clarity).

The compositions of the hydrogen bonds between adjacent cyclo­butanes are defined by the orientations (*i.e. syn*, *anti*, *gauche*) of the OH groups of the phenols. Specifically, a primary six-membered ring of the **hcb** subnet involves nodes with hydrogen bonds of alternating two *anti* orientations [11.53 Å (**CB1**), 11.49 Å (**CB2**)] and one *syn* orientation (12.15 Å). The phenol groups in the *gauche* (11.81 Å) orientation interconnect the twofold interpenetrated **hcb** subnets [orange, Fig. 6 ▸(*c*)] and complete the self-catenation. A secondary six-membered ring is generated from interconnection of the **hcb** subnets (Fig. 7 ▸). The secondary six-membered ring involves nodes with hydrogen bonds of alternating two *anti* orientations (**CB1, CB2**) and one *gauche* orientation. Additionally, eight-membered rings are generated from the interconnection of three **hcb** subnets, involving nodes of alternating one *anti* (**CB1** or **CB2**), one *gauche* and one *syn* orientation.

The **mok** framework of **1a** also exhibits the overall twofold interpenetration [Fig. 6 ▸(*d*)]. Highly disordered electron density consistent with MeOH as solvent is located in lacunae (∼180 Å^3^) (Spek, 2015 ▸) at the intersection of the interpenetrated **hcb** and **mok** subnets and nets, respectively. The **mok** net of **1a** was, before now, an unrealized network in structures of purely organic solids.

### 2.7. A **mok** net purely organic in origin   

The **mok** net **1a** represents a rare family of entanglements that have only been realized in coordination polymers and metal–organic frameworks. For metal–organic materials, the metal centers and organic linkers are nodes and bridges, respectively, Gong *et al.*, 2011 ▸; Liang *et al.*, 2013 ▸; Zhang *et al.*, 2015 ▸). O’Keeffe has pointed out that a single **mok** net can be considered difficult to achieve chemically given that one distance between two nodes is shorter than the distance between linked nodes. The short distance of a **mok** net corresponds to two nodes between the interpenetrated **hcb** layers. For **1a** the corresponding distances are 10.1 Å (non-linked nodes) and 11.5 Å (linked nodes); however, we note that here the twofold interpenetration generates much shorter distances between nodes of two separate nets of the interpenetrated structure (*i.e.* 6.07, 6.38 Å).

A major factor that defines how **1a** supports the formation of the **mok** net relates to the different orientations that the cyclo­butane assumes to define the nodes and edges of the network. Two copies of the same molecule that are present in two different conformations (*i.e.* **CB1** and **CB2**) self-assemble to form the network. The conformations support the four different types of linkages (*i.e. anti* (2), *syn*, *gauche*) to create six- and eight-membered rings. In doing so, the cyclo­butanes for both the six- and eight-membered rings act as either double hydrogen-bond donors (DD), double hydrogen-bond acceptors (AA) or a donor/acceptor (DA) (Table 2 ▸, Fig. 7 ▸). The AA linkages involve acceptor pyridyls in the 3,4-position of the cyclo­butane ring, whereas the acceptors of the DA linkages are fixed in either the 3-position (3-acceptor) or 4-position (4-acceptor) of the ring. The cyclo­butane **1a** effectively adapts to conform to the topology of the **mok** net by using chemical information stored at the molecular level (*i.e.* cyclo­butane and conformation) that is then expressed as required at the supramolecular (*i.e.* hydrogen-bond donor and acceptor capacities) level.

## 3. Conclusions   

We have reported the first **mok** network composed of purely organic components. The cyclo­butane **1a** contains a combination of tetrahedrally disposed (Zhang *et al.*, 2012 ▸) hydrogen-bond donor and acceptor sites synthesized in the solid state using a newly developed supramolecular protecting-group strategy. Hydroxyl donor sites, which add a second degree of flexibility, are used to achieve the unique topology. We believe our observation of the building block **1a** to support different network linkages to form the **mok** net serves as an important example on how to achieve supramolecular complexity from redundant molecular information. The fact that the solid state can be exploited for such design, particularly given the high degree of control of directionality for covalent bond formation, can be expected to encourage further work in the field.

## 4. Related literature   

The following references are cited in the supporting information: Sheldrick (2015*a* ▸,*b* ▸); Spek (2003 ▸); Blatov *et al.* (2016 ▸, 2010 ▸); Alexandrov *et al.* (2011 ▸); Kraus & Nolze (1996 ▸).

## Supplementary Material

Crystal structure: contains datablock(s) mcg16180lt, mcg16122, web134, web006, mcg16113. DOI: 10.1107/S2052252519011382/yc5020sup1.cif

Structure factors: contains datablock(s) web006. DOI: 10.1107/S2052252519011382/yc5020sup2.hkl

Structure factors: contains datablock(s) web134. DOI: 10.1107/S2052252519011382/yc5020sup3.hkl

Structure factors: contains datablock(s) mcg16113. DOI: 10.1107/S2052252519011382/yc5020sup4.hkl

Structure factors: contains datablock(s) mcg16122. DOI: 10.1107/S2052252519011382/yc5020sup5.hkl

Structure factors: contains datablock(s) mcg16180lt. DOI: 10.1107/S2052252519011382/yc5020sup6.hkl

Supporting information file. DOI: 10.1107/S2052252519011382/yc5020sup7.pdf

CCDC references: 1419186, 1419187, 1832096, 1832097, 1832098

## Figures and Tables

**Table 1:** Selected hydrogen-bond distances and angles

Molecule	Distance (Å)	O—H⋯N angle (°)
**CB1**		
O1(*anti*)⋯N4	2.764 (3)	163.96 (3)
O2(*gauche*)⋯N3	2.676 (3)	167.71 (3)
		
**CB2**		
O3(*syn*)⋯N1	2.812 (3)	174.94 (3)
O4(*anti*)⋯N2	2.733 (3)	166.49 (3)

**Table 2:** Unique rings of **1a** **mok** network

No.	Cyclo­butane	Overall type†	Donor type	Acceptor type
Six-membered rings				
Primary				
1	CB2	DD	*syn*, *anti*	–
2	CB1	DA	*anti*	3-acceptor
3	CB2	DA	*syn*	3-acceptor
4	CB1	DA	*anti*	4-acceptor
5	CB2	DA	*anti*	4-acceptor
6	CB1	AA	–	3,4-acceptor
Secondary				
7	CB1	DD	*anti*/*gauche*	–
8	CB2	DA	*anti*	4-acceptor
9	CB1	DA	*gauche*	4-acceptor
10	CB2	DA	*anti*	3-acceptor
11	CB1	DA	*anti*	3-acceptor
12	CB2	AA	–	3,4-acceptor
				
Eight-membered rings				
1,5	CB1	DD	*anti*, *gauche*	–
2,6	CB2	DA	*syn*	4-acceptor
3,7	CB1	DA	*gauche*	3-acceptor
4,8	CB2	AA	–	3,4-acceptor

† Cyclo­butane participation in six- and eight-membered rings as AA = double hydrogen-bond acceptors, DD = double hydrogen-bond donors, or DA = hydrogen-bond donor and acceptor.
