# Supplementary material for: Diversifying molecular and topological space via a supramolecular solid-state synthesis: a purely organic mok net sustained by hydrogen bonds
Source: IUCrJ. 2019 Sep 7;6(Pt 6):1032–9. doi: 10.1107/S2052252519011382 (PMC6830215; doi:10.1107/S2052252519011382)
Supplement: Supplementary file 7 [file m-06-01032-sup7.pdf]

IUCrJ

Volume 6 (2019)

Supporting information for article:

Diversifying Molecular and Topological Space via a Supramolecular Solid-State Synthesis: A Purely Organic mok Net Sustained by Hydrogen Bonds

Shalisa M. Oburn, Michael A. Sinnwell, Devin P. Ericson, Eric W. Reinheimer, Davide M. Proserpio, Ryan H. Groeneman and Leonard MacGillivray

S1. General Methods and Materials

All solvents used were available from commercial sources and were used without any further purification. Ethanol (95%), dichloromethane (99.5%) and anhydrous ethyl acetate (99.8%) were purchased from Sigma Aldrich (St. Louis, MO, USA).

S2. Synthesis of *trans*-1-(4-phenol)-2-(4-pyridyl) ethylene (1b)

The reactant **1b** was prepared as reported.[1] Single crystals of **1b** were grown by slow evaporation from a 1:1 (v:v) methanol and ethyl acetate solution over a period of 10 d. ^{1}H NMR (300 MHz, DMSO-d_6): δ 9.76 (s, 1H), 8.49 (d, *J* = 6.0 Hz, 2H), 7.57 – 7.33 (m, 5H), 7.00 (d, *J* = 16.4 Hz, 1H), 6.86 – 6.70 (m, 2H).

S3. Synthesis of the co-crystal (diI-res)·2(1c)

The reactive olefin *trans*-1-(4-acetoxy)-2-(4-pyridyl) ethylene (**1c**) was prepared via a previously published method.[1] Single crystals of **1c** were grow by dissolving 25 mg in 2 mL of ethyl acetate and upon slow evaporation crystals suitable for X-ray diffraction were formed overnight. ^{1}H NMR (300 MHz, DMSO-d_6): δ 8.55 (dd, *J* = 4.6, 1.5 Hz, 2H), 7.75 – 7.62 (m, 2H), 7.55 (dt, *J* = 5.7, 5.2 Hz, 3H), 7.28 – 7.11 (m, 3H), 2.28 (s, 3H). Cocrystals of (**diI-res**)·2(**1c**) before photoreaction was obtained *via* the slow evaporation method at ambient conditions based upon combining 50 mg (0.21 mmol) of **1c** dissolved in 3 mL of ethyl acetate with 56 mg (0.16 mmol) of 4,6-diiodoresorcinol in 2 mL of ethanol. Suitable crystals for single-crystal X-ray diffraction were formed after 2 d of slow evaporation. Colorless crystals were removed from the vial and allowed to dry in air; yield 0.089 g (84%). ^{1}H NMR (300 MHz, DMSO-d_6): δ 10.40 (s, 2H), 8.55 (d, *J* = 5.8 Hz, 4H), 7.77 (s, 1H), 7.69 (d, *J* = 8.6 Hz, 4H), 7.60 – 7.49 (m, 6H), 7.20 (t, *J* = 13.2 Hz, 6H), 6.58 (s, 1H), 2.28 (s, 6H).

S4. Photodimerization

Crystals were finely ground using a mortar and pestle and then spread thinly between a pair of Pyrex glass plates. The samples were irradiated in 6 hr. intervals (ACE Glass photochemistry cabinet, 450 W medium-pressure Hg lamp). Cyclobutane formation was monitored by using ^{1}H NMR spectroscopy. Various attempts to achieve a single crystal-to-single crystal reaction were performed; however in each case crystallinity was lost after the initial 6 hr. of UV exposure.

S5. Synthesis of the co-crystal (diI-res)·(1d)

The cyclobutane *rctt*-1,2-bis(4-pyridyl)-3,4-bis(4-acetoxyphenyl)cyclobutane (**1d**) was generated in quantitative yield within 100 hrs. of exposure of a finely ground crystalline sample to UV light. In

order to confirm the stereochemistry of the photoproduct, single crystals were obtained by recrystallization from 4 mL of an ethanol and ethyl acetate (1:1, v:v) solution. Slow evaporation of the solvents at ambient conditions over a period of 3 d afforded small crystals suitable for X-ray diffraction analysis. ^{1}H NMR (300 MHz, DMSO-d_6): δ 10.36 (s, 2H), 8.33 (d, J = 6.0 Hz, 4H), 7.76 (s, 1H), 7.33 – 7.11 (m, 8H), 6.91 (d, J = 8.6 Hz, 4H), 6.57 (s, 1H), 4.58 (s, 4H), 2.19 (s, 6H).

S6. Isolation of *rctt*-1,2-bis(4-pyridyl)-3,4-bis(4-phenol)cyclobutane (1a)

The photoproduct **1a** was isolated *via* one-step removal of the acetyl group and resorcinol template. The photoreacted co-crystal (**diI-res**)·(**1d**) (104.5 mg) was ground with NaOH pellets (173.8 mg, 4.35 mmol) with the addition of 2 drop of water for 10 minutes using a agate mortar and pestle. The slurry was diluted with water (30 mL), acidified to with aqueous HCl to pH 2, and washed with dichloromethane (3 x 5 mL). The resulting aqueous solution was heated for 10 minutes to remove residual dichloromethane. Upon cooling, the aqueous solution was neutralized with NaOH and the resulting precipitate was filtered to yield **1a** (36.7 mg, 74.7 %). Single crystals of (**1a**)·0.5(**MeOH**) were grown by slow evaporation from an aqueous methanol solution over 5 d.^{1}H NMR (300 MHz, DMSO-d_6): δ 9.08 (s, 2H), 8.30 (d, J = 6.0 Hz, 4H), 7.23 – 7.11 (m, 4H), 6.96 (d, J = 8.5 Hz, 4H), 6.53 (d, J = 8.5 Hz, 4H), 4.43 (d, 7.2 Hz, 2H), 4.34 (d, 7.2 Hz, 2H). ^{13}C NMR (100 MHz, DMSO-d_6): δ 155.40, 149.42, 149.05, 130.11, 129.04, 123.46, 114.73, 45.89, 45.05.

S7. ^{1}H NMR spectroscopy

All ^{1}H NMR and ^{13}C NMR spectra were obtained in DMSO-d_6 on a Bruker Fourier-300 NMR spectrometer operating at 300 MHz and 100 MHz, respectively. All data were processed with MestReNova 6.0.2 software program.

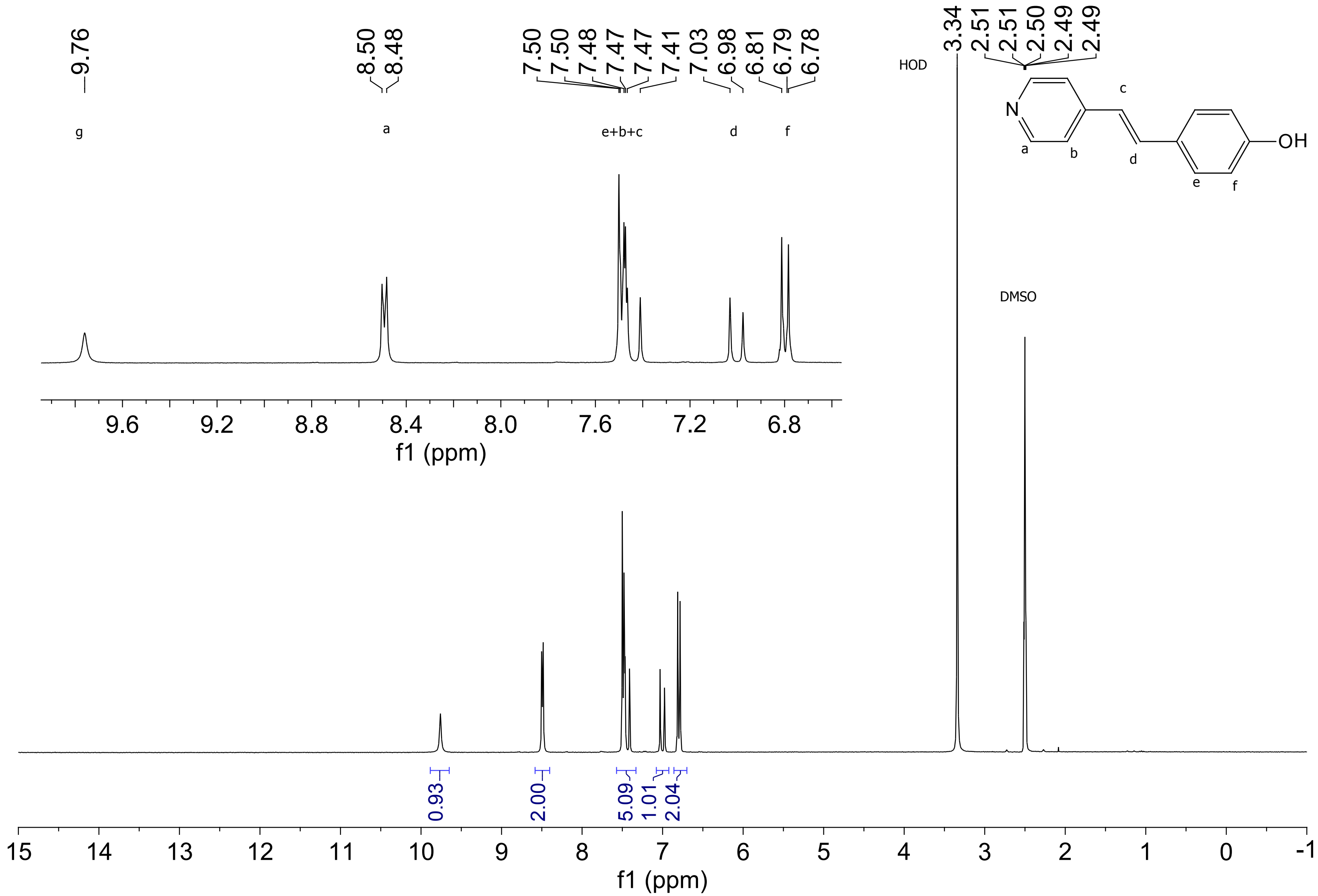

Figure S1 ^{1}H NMR spectrum of synthesized **1b** after 50 hr of UV exposure.

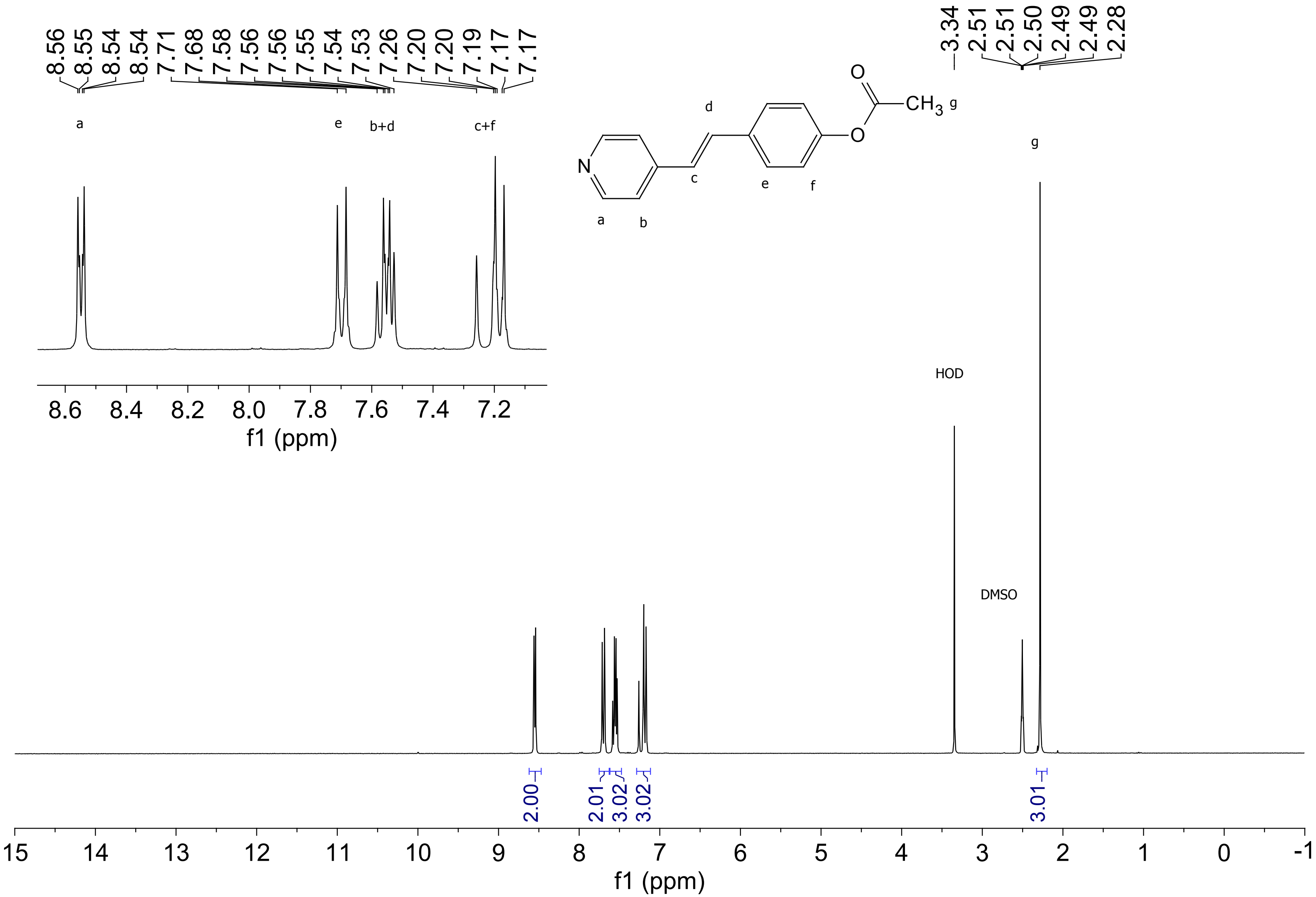

Figure S2 ^{1}H NMR spectrum of synthesized **1c** after 50 hr of UV exposure.

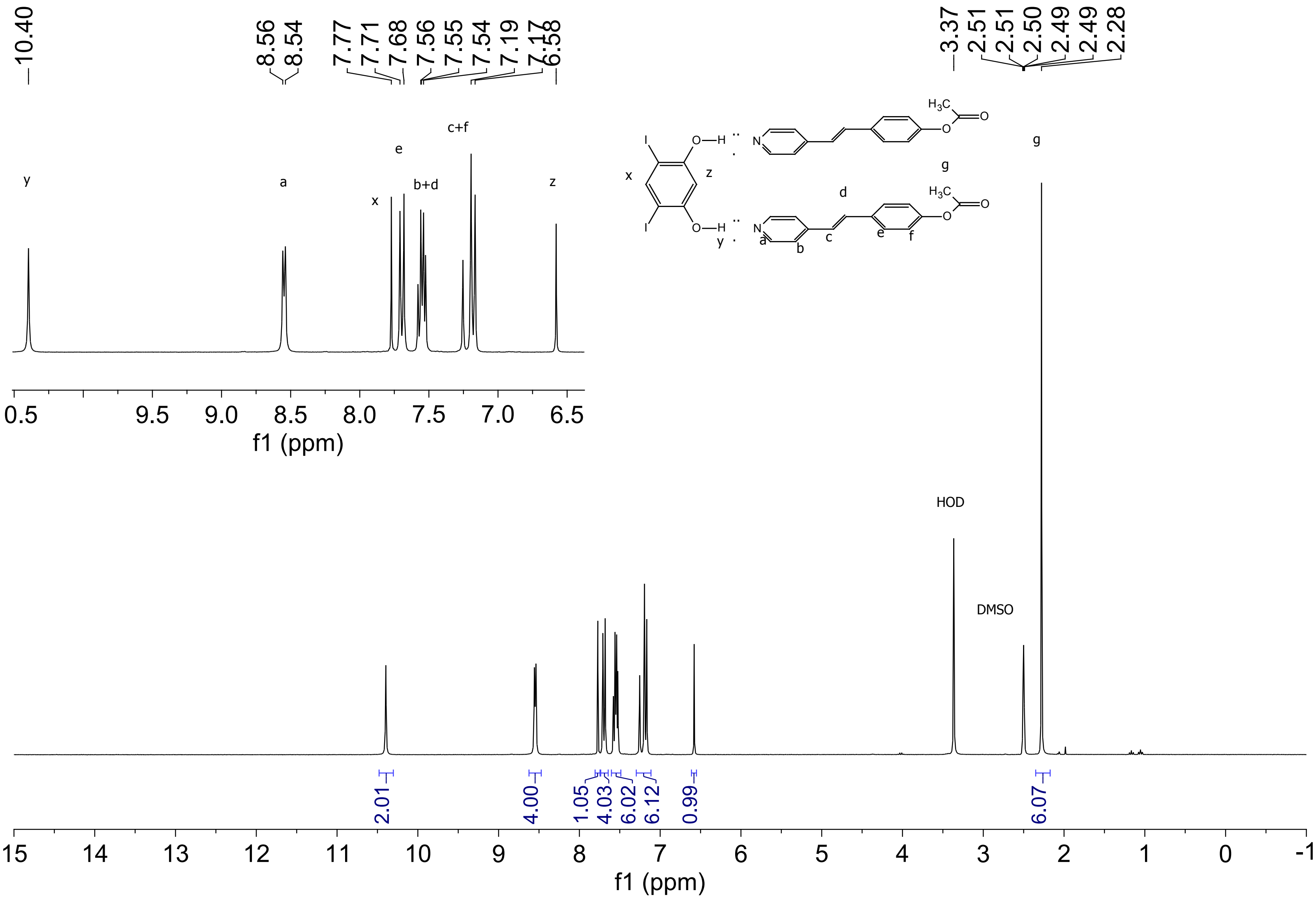

Figure S3 ^{1}H NMR spectrum of the co-crystal (**diI-res**)·2(**1c**) before UV exposure.

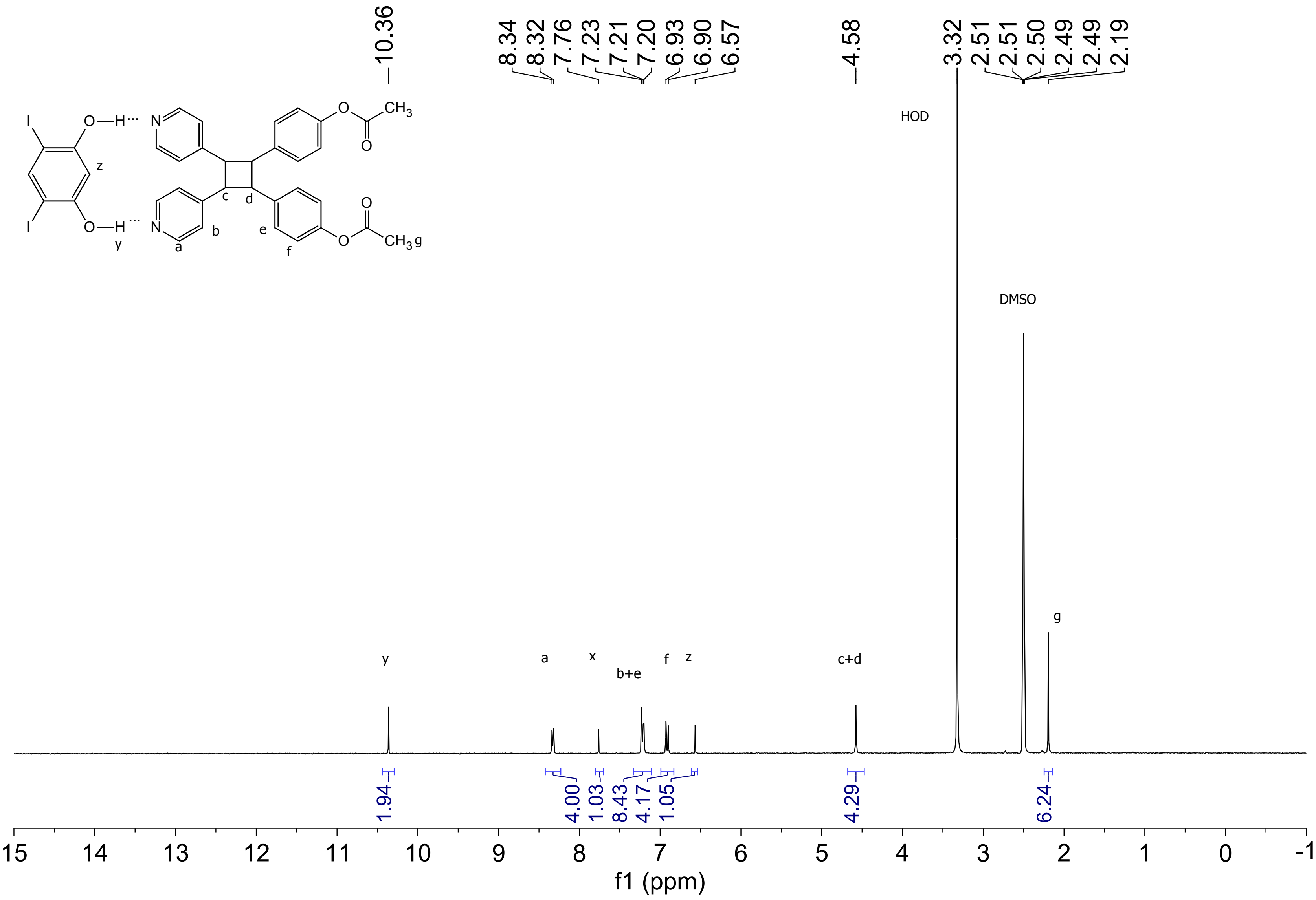

Figure S4 ^{1}H NMR spectrum of the co-crystal (**diI-res**)·2(**1d**) after UV exposure.

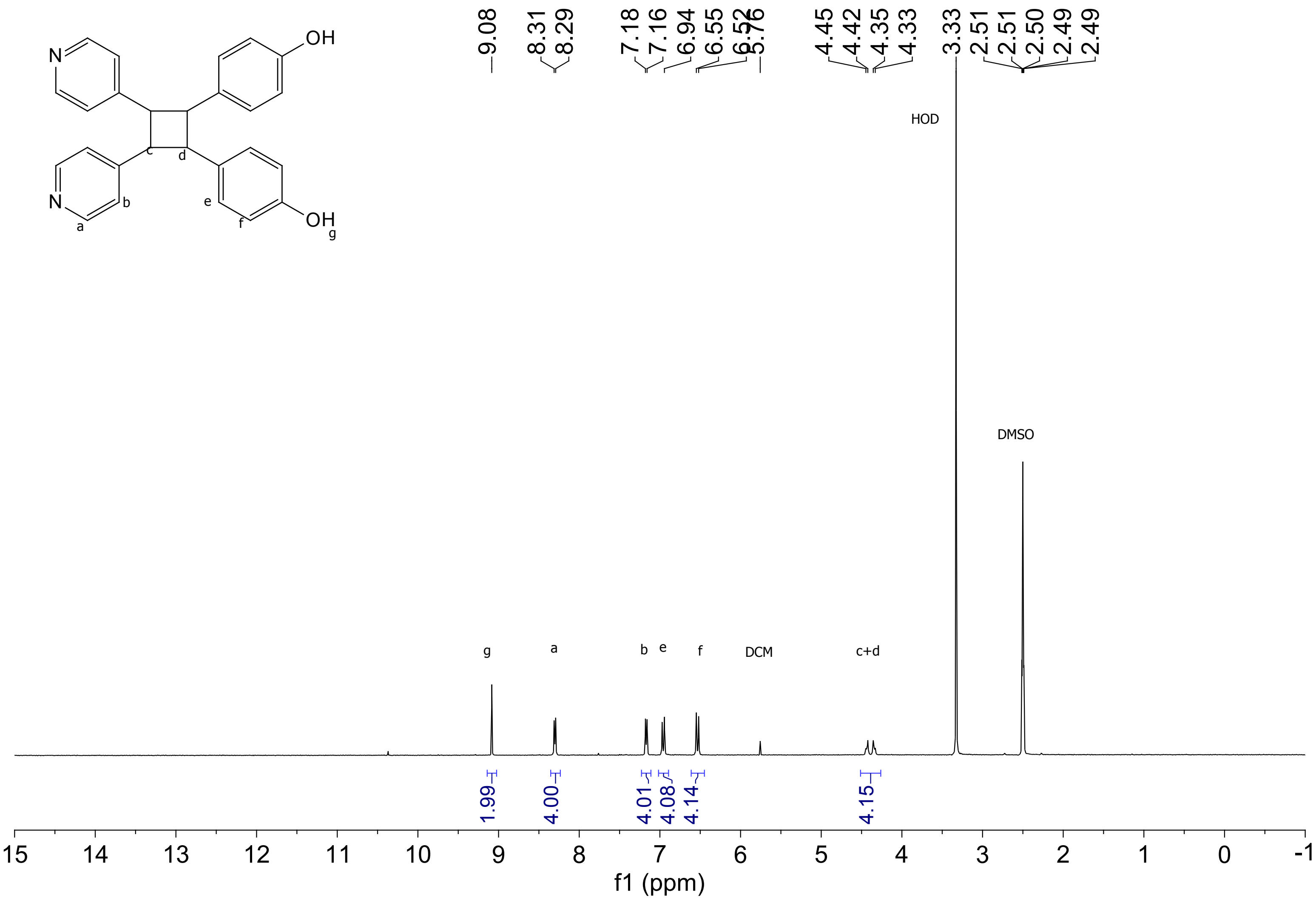

Figure S5 ^{1}H NMR spectrum of isolated photoproduct **1a**. DCM corresponds to residual dichloromethane.

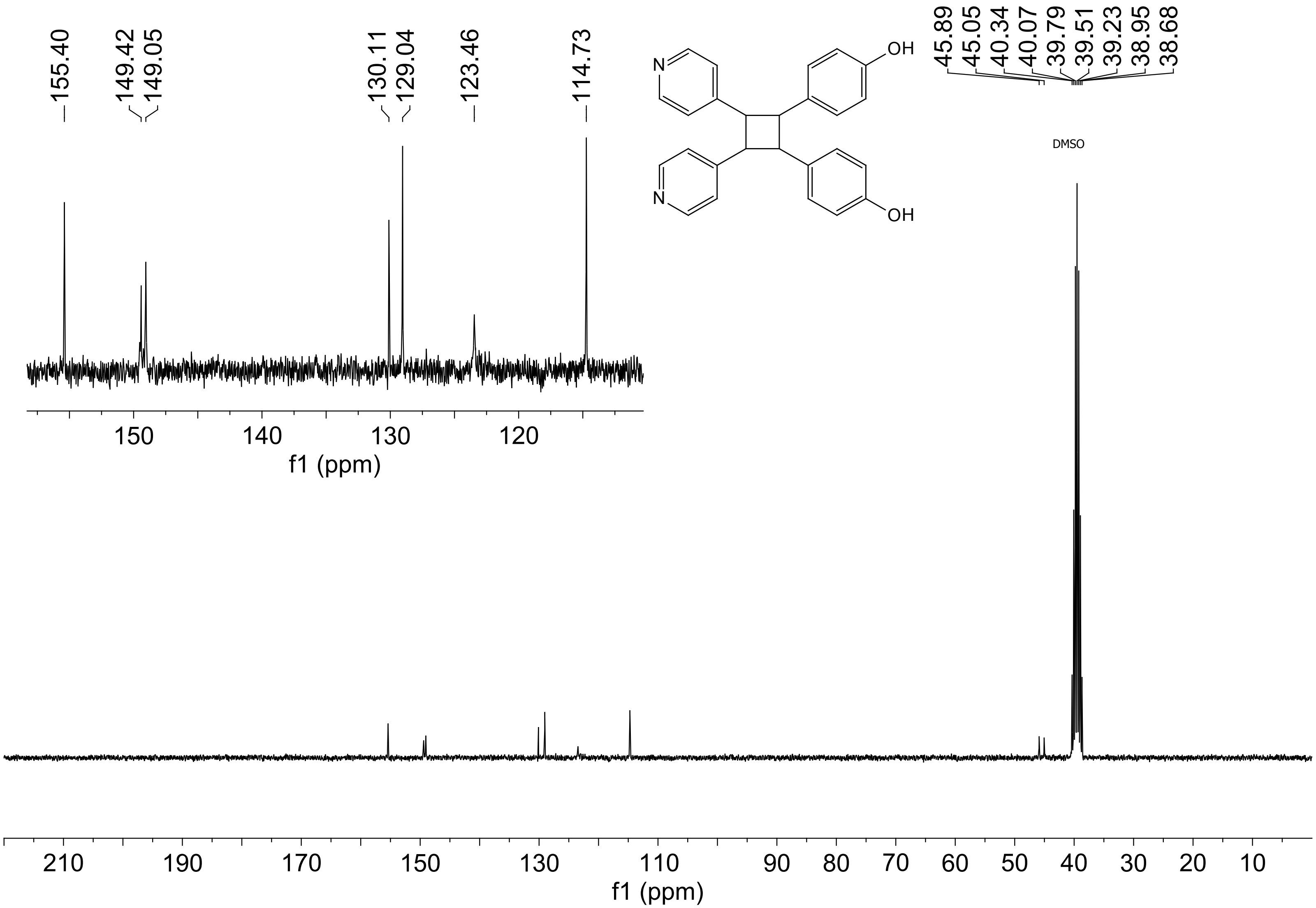

Figure S6 ^{13}C NMR spectrum of isolated photoproduct **1a**.

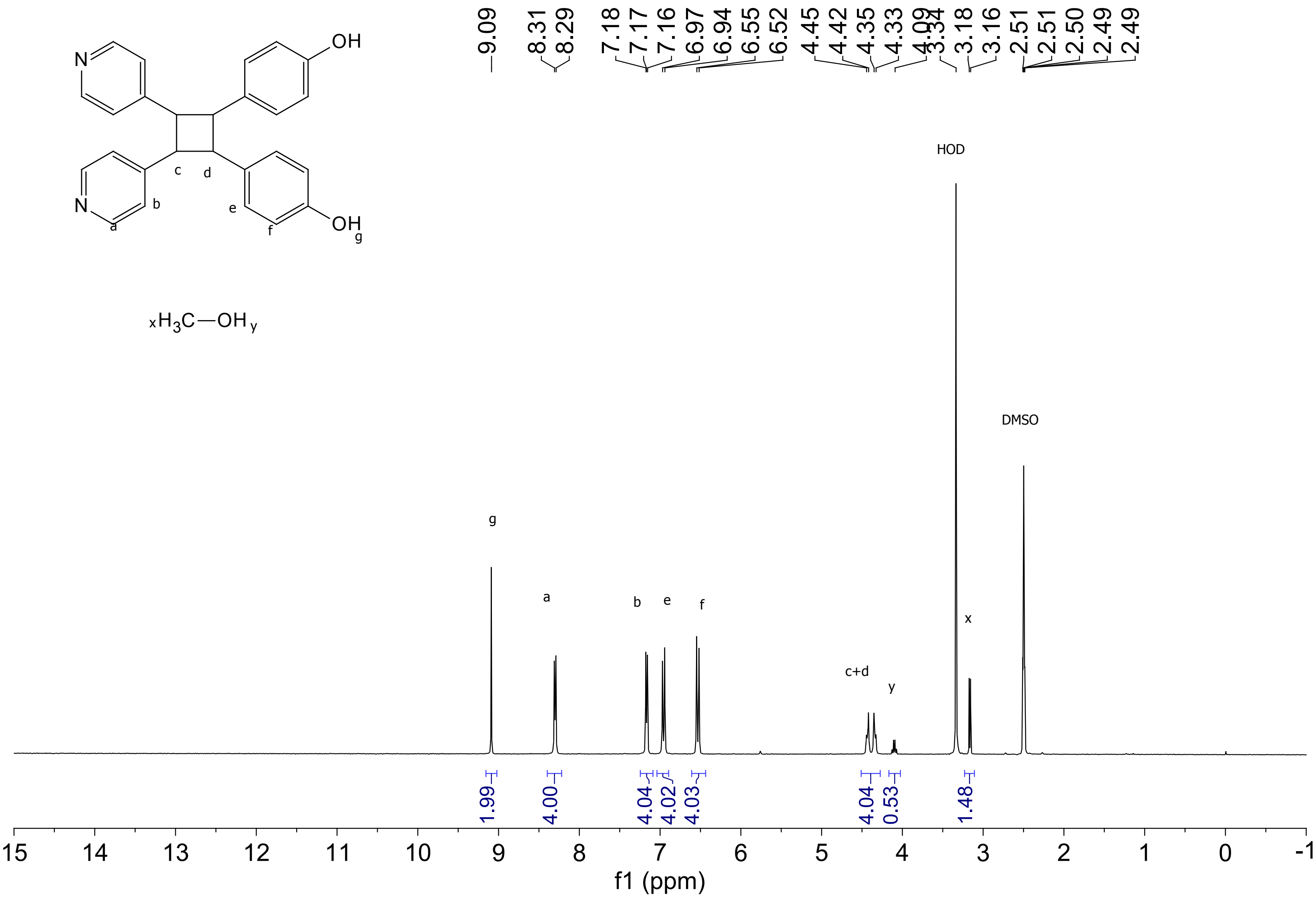

Figure S7 ^{1}H NMR spectrum of isolated photoproduct from an aqueous methanol solution, (**1a**)·**0.5**(**MeOH**).

S8. Single-crystal X-ray diffraction measurements

Diffraction data for **1b**, **1c**, (**diI-res**)·2(**1c**), and (**1a**)·0.5(**MeOH**) were collected on a Bruker Neditonius APEX II Kappa single-crystal X-ray diffractometer at either 293 or 190 K using graphite-monochromated Mo $K_{\alpha 1}$ radiation (λ = 0.71073 Å). Structure solution and refinement were accomplished using ShelXT (Sheldrick, 2015*a*) and ShelXL (Sheldrick, 2015*b*), respectively. The structure was solved using direct methods. All non-hydrogen atoms were identified from the difference Fourier map and refined anisotropically. All hydrogen atoms were placed in their calculated positions and were refined using isotropic thermal parameters. The hydroxyl hydrogen atoms of (**1a**)·0.5(**MeOH**) of the cyclobutanes were identified from the difference Fourier map and refined. In the X-ray refinement of (**1a**)·0.5(**MeOH**), guest MeOH molecules exhibited high thermal disorder and the final structure model was refined excluding solvent MeOH molecules using SQUEEZE routine of PLATON (Spek, 2003). SQUEEZE output indicated a solvent accessible void volume of 181 $Å^3$ and an electron count of 15. The presence of a stoichiometric amount methanol was determined using ^{1}H NMR on a single crystal of (**1a**)·0.5(**MeOH**).

Due to the small size of the single crystal, (**diI-res**)·2(**1d**) was collected using synchrotron radiation. Intensity data were collected at 150 K on a D8 goniostat equipped with a Bruker APEXII CCD detector at Beamline 11.3.1 at the Advanced Light Source (Lawrence Berkeley National Laboratory) at a wavelength of 1.2399 Å. Data collection frames were measured for a duration of 1 second at 0.3^o intervals of ω with a maximum 2θ value of ~60°. Again the structure solution and refinement were performed using the SHELX suite of programs. The structure was solved using direct methods and all non-hydrogen atoms were identified and were refined anisotropically. Lastly, hydrogen atoms were again placed in their calculated positions and were refined isotropically. This synchrotron-based crystallographic data were collected through the SCrALS (Service Crystallography at Advanced Light Source) program at the Small-Crystal Crystallography Beamline 11.3.1 at the Advanced Light Source. Crystallographic parameters for each structure are listed in **Table 1**.

Table S1 Crystallographic and refinement data for **1b**, **1c**, and (**diI-res**)·2(**1c**).

Compound	1b	1c	(diI-res)·2(1c)
**CCDC code**	1832096	1832097	1419187
**Formula**	$C_{13}H_{11}NO$	$C_{15}H_{13}NO_2$	$C_{36}H_{30}I_2N_2O_6$
**Formula weight**	197.23	239.26	840.42
**Temp.**	190(2)	190(2)	293(2)
**Space group**	$Pca2_1$	*Pbca*	*P-1*
**a, Å**	23.354(3)	10.5366(11)	9.3954(9)
**b, Å**	5.6449(7)	7.4255(7)	10.055(2)
**c, Å**	7.7373(10)	31.091(3)	19.911(2)
**α, deg.**	90	90.00	101.278(5)
**β, deg.**	90	90.00	92.285(5)
**γ, deg.**	90	90.00	111.552(5)
**volume, Å^3**	1020.0(2)	2432.5(4)	1702.8(4)
**Z**	4	8	2
**Density (calculated), g/cm^3**	1.284	1.307	1.639
**μ, mm^{-1}**	0.082	0.087	1.894
**Scan**	ω and φ scans	*ω* and φ scans	φ scan
**θ range for data collection, deg.**	3.158-23.226	2.335-25.245	1.051-27.946
**Reflections measured**	11876	30658	14414
**Independent observed reflns.**	1384	2205	8137
**Independent reflns. [I>2σ]**	866	1517	4839
**Data/restraints/parameters**	1384/1/138	2205/0/215	8137/0/420

**R_{int}**	0.0623	0.0719	0.0333
**Final R Indices [I>2σ]**	R_1 = 0.0414,	R_1 = 0.0565,	R_1 = 0.0369,
	$wR2$ = 0.0640	$wR2$ = 0.1605	$wR2$ = 0.0906
**R Indices (all data)**	R_1 = 0.0938,	R_1 = 0.0917,	R_1 = 0.0834,
	$wR2$ = 0.0757	$wR2$ = 0.1800	$wR2$ = 0.1199
**Goodness-of-fit on F^2**	1.018	1.087	1.017

Table S2 Crystallographic and refinement data for (**diI-res**)·2(**1d**) and (**1a**)·0.5(**MeOH**).

Compound	(diI-res)·2(1d)	(1a)·0.5(MeOH)
**CCDC code**	1419186	1832098
**Formula**	$C_{36}H_{30}I_2N_2O_6$	$C_{26}H_{22}N_2O_2$
**Formula weight**	840.42	394.45
**Temp.**	150(2)	190(2)
**Space group**	*P2₁/c*	*I2/a*
**a, Å**	21.7663(10)	19.7244(19)
**b, Å**	9.8526(5)	12.3920(12)
**c, Å**	16.2690(7)	35.750(3)
**α, deg.**	90.00	90.00
**β, deg.**	102.198(2)	99.796(6)
**γ, deg.**	90.00	90.00
**volume, Å^3**	3410.2(3)	8610.8(14)
**Z**	4	16
**Density (calculated), g/cm^3**	1.637	1.217
**μ, mm^{-1}**	8.277	0.077
**Scan**	*ω* scan	*ω* and φ scans
**θ range for data collection, deg.**	3.976-48.092	2.173-25.251
**Reflections measured**	23281	66161
**Independent observed reflns.**	6120	7778
**Independent reflns. [I>2σ]**	5506	5946
**Data/restraints/parameters**	6120/3/395	7778/29/569

**R_{int}**	0.0552	0.0450
**Final R Indices [I>2σ]**	R_1 = 0.0590,	R_1 = 0.0566,
	$wR2$ = 0.1651	$wR2$ = 0.1402
**R Indices (all data)**	R_1 = 0.0627,	R_1 = 0.0756,
	$wR2$ = 0.1684	$wR2$ = 0.1485
**Goodness-of-fit on F^2**	1.048	1.047

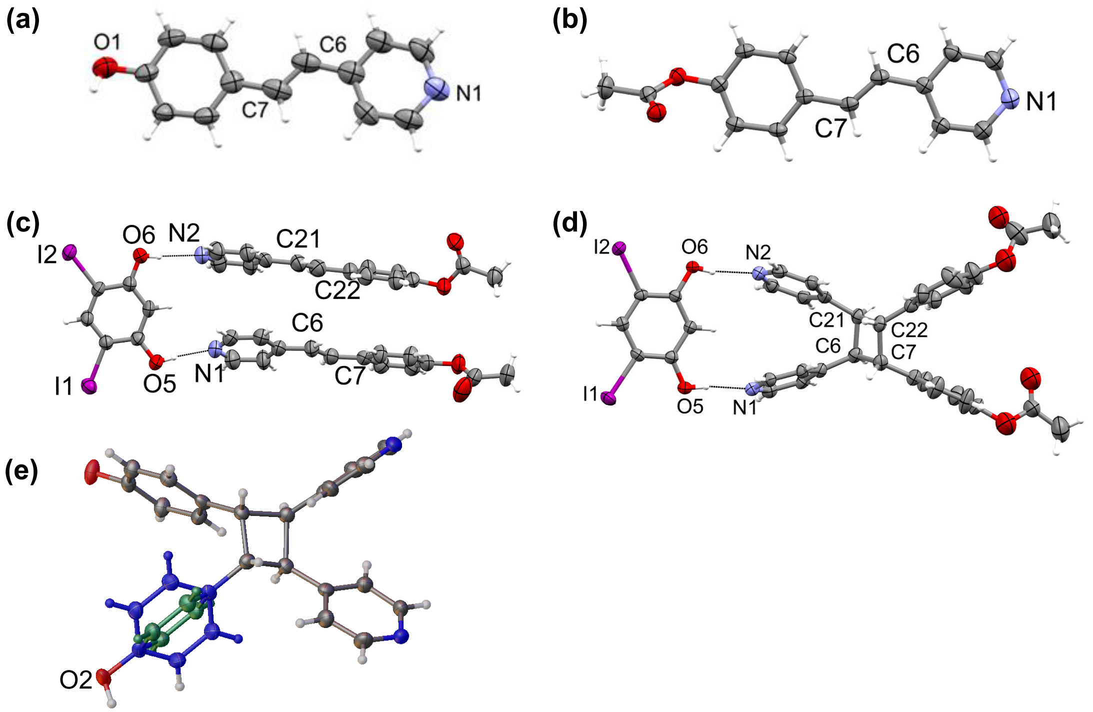

Figure S8 ORTEP with thermal ellipsoids set at 50 % probability for (a) **1b**, (b) **1c**, (c) **(diI-res)**·2(**1c**), (d) **(diI-res)**·(**1d**), and (e) **CB1** of (**1a**)·0.5(**MeOH**) showing disorder of phenyl ring corresponding to *gauche*-hydroxyl orientation (site A: 0.90(1), green; site B: 0.10(1), blue).

S9. ToposPro calculations:

The network simplification of (**1a**)·0.5(**MeOH**) was performed with ToposPro software (Blatov, 2014). The *Auto CN* (Blatov, 2016) and *ADS* (Blatov et al., 2011; Blatov et al., 2010) commands upon choosing the cyclobutanes as nodes gave the simplified net with $6^5.8$-**mok** topology. ToposPro also indicated an identical **mok** net related by a an inversion center (-1). Below is part of the ToposPro output, where ZA1 and ZA2 represent the barycenter of the two unique cyclobutanes **CB1** and **CB2**:

```
Topology for ZA1
--------------------
Atom ZD1 links by bridge ligands and has
Common vertex with                     R(A-A)
ZD 2    0.0806    0.9948    0.3779   ( 0 1 0)    11.494A
ZD 2    1.0806    0.9948    0.3779   ( 1 1 0)    11.529A
ZD 2    0.9194    0.4948    0.1221   ( 0 1 0)    11.815A
ZD 2    0.4194   -0.0052    0.6221   (-1 0 0)    12.152A
Topology for ZD2
--------------------
Atom ZD2 links by bridge ligands and has
Common vertex with                     R(A-A)
ZD 1   -0.0746    0.9822    0.1381   ( 0 1 0)    11.494A
ZD 1    0.9254    0.9822    0.1381   ( 1 1 0)    11.529A
ZD 1    0.0746    0.4822    0.3619   (-1 1 0)    11.815A
ZD 1    0.5746   -0.0178   -0.1381   (-1 0-1)    12.152A
-------------------------
Structure consists of 3D framework with ZA
There are 2 interpenetrating nets
FISE: Full interpenetration symmetry elements
---------------------------------------------
1: -1
---------------------------------------------
PIC: [0,1,0][1,0,0][1/2,1/2,1/2] (PICVR=1)
Zt=1; Zn=2
Class IIa  Z=2
Coordination sequences
----------------------
ZA1:  1  2  3   4   5   6   7   8    9   10
Num   4 12 30  66 107 154 211 278  353  438
Cum   5 17 47 113 220 374 585 863 1216 1654
```

```
----------------------
ZA2:  1  2  3   4   5   6   7   8    9   10
Num   4 12 30  66 107 154 211 278  353  438
Cum   5 17 47 113 220 374 585 863 1216 1654
----------------------
TD10=1654
Vertex symbols for selected sublattice
--------------------------------------
ZA1 Point symbol:{6^5.8}
Extended point symbol:[6.6.6.6.6(2).8(4)]
--------------------------------------
ZA2 Point symbol:{6^5.8}
Extended point symbol:[6.6.6.6.6(2).8(4)]
--------------------------------------
```

Point symbol for net: {6^5.8}
4-c net; uninodal net

Topological type: **mok**; sev-4-Cccm (uninodal.ttd)

S10. Powder X-ray diffraction measurements

All powder samples were mounted on glass slides. PXRD data were collected by a Siemens D5000 X-ray diffractometer using Cu Kα_1 radiation (λ = 1.54056 Å) (scan type: locked coupled; scan mode: continuous; step size: 0.02°). Simulated powder patterns were generated and refined to experimental powder patterns using *PowderCell* 2.3 (Kraus & Nolze, 1996).

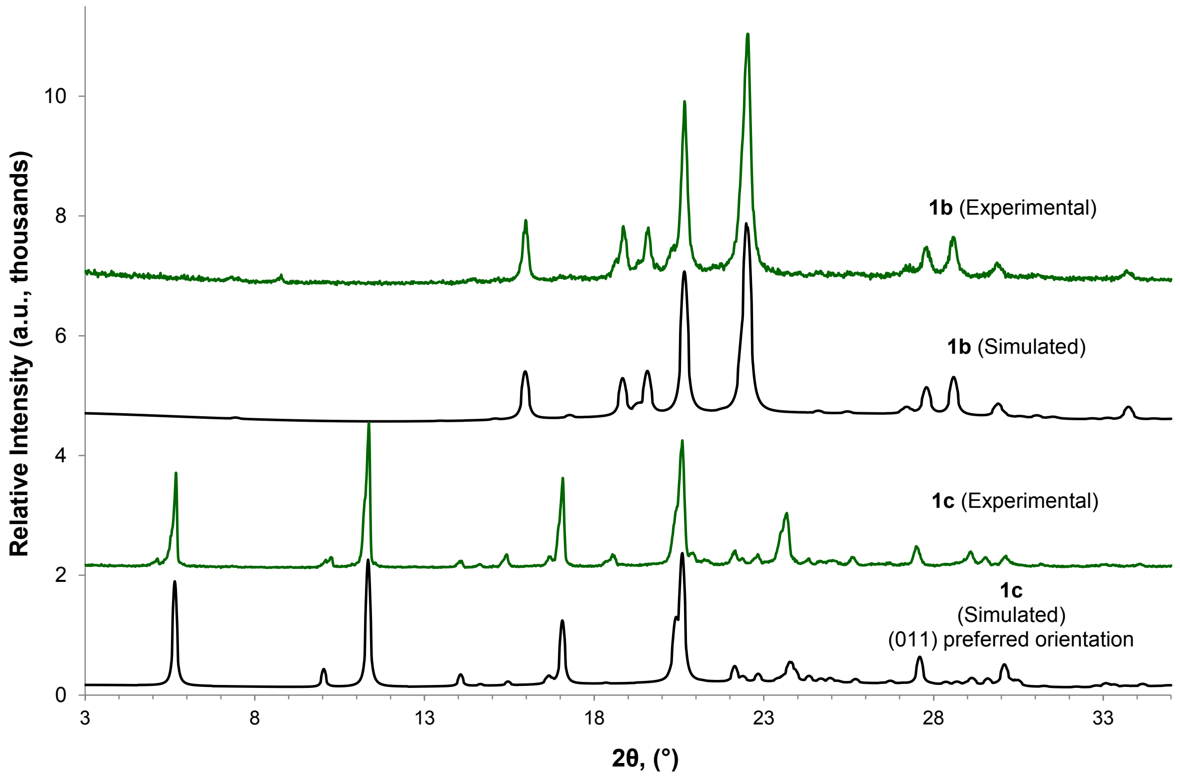

Figure S9 Powder X-ray diffraction of unprotected olefin **1b** and protected olefin **1c** powders (green) compared simulated pattern from single-crystal X-ray diffraction data (black).

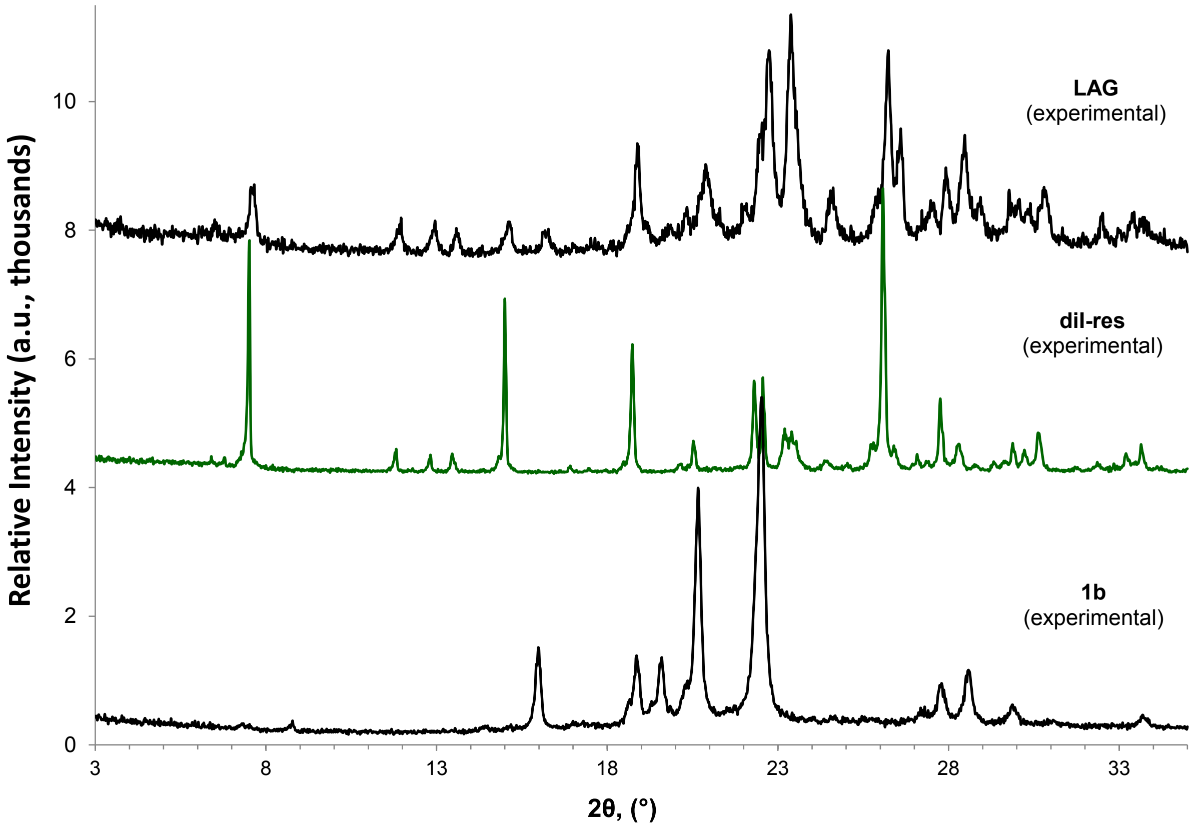

Figure S10 Powder X-ray diffraction of liquid-assisted grinding experiments (LAG) of unprotected **1b** with **res** template **diI-res** using ethanol (black, top) compared to single component patterns of **diI-res** (green, middle) and **1b** (black, bottom).

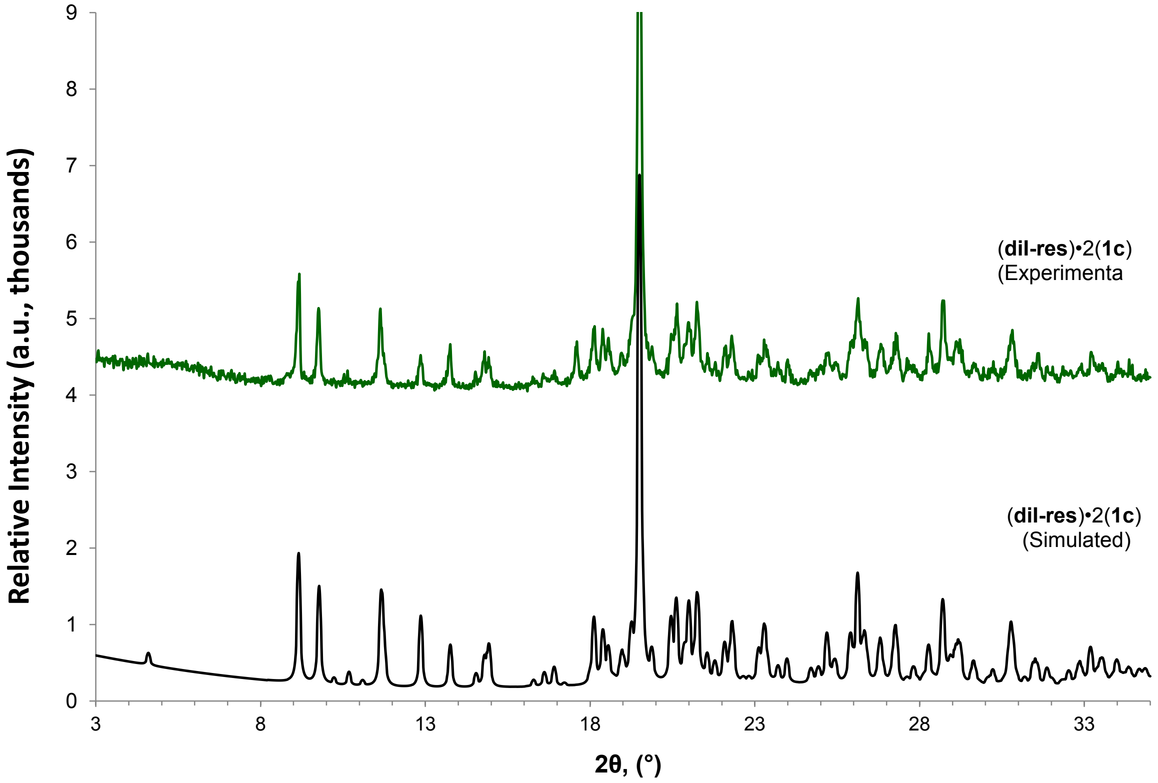

Figure S11 Powder X-ray diffraction of cocrystal of (**diI-res**)·2(**1c**) powder (green, top) compared simulated pattern from single-crystal X-ray diffraction data (black, bottom).

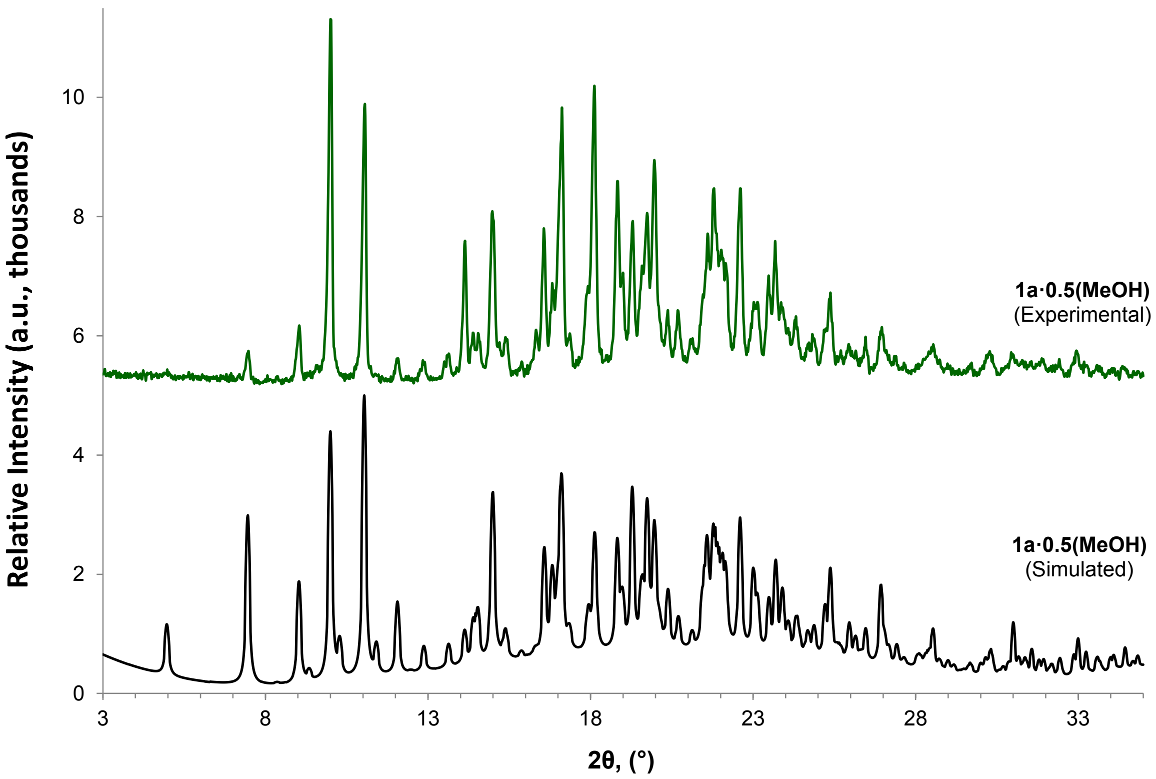

Figure S12 Powder X-ray diffraction of (**1a**)·0.5(**MeOH**) (green, top) and simulated pattern from single-crystal X-ray diffraction data (black, bottom).

S11. References

1. S. Yin, H. Sun, Y. Yan, H. Zhang, W. Li and L. Wu, *J. Colloid Interface Sci.*, **2011**, *361*, 548.
2. G. Sheldrick, *Acta Crystallogr. Sect. A*, **2015**, *71*, 3.
3. G. Sheldrick, *Acta Crystallogr. Sect. C*, **2015**, *71*, 3.
4. A. L. Spek, *J. Appl. Crystallogr.*, **2003**, *36*, 7.
5. Blatov V. A., Shevchenko A.P., Proserpio D.M. *Cryst. Growth Des*., **2014**, *14*, 3576.
6. Blatov V. A. *Struct. Chem*., **2016**, *27*, 1605.
7. Alexandrov E.V., Blatov V.A., Kochetkov A.V., Proserpio D.M. *CrystEngComm*, **2011**, *13*, 3947.
8. Blatov V.A., O'Keeffe M., Proserpio D. M. *CrystEngComm*, **2010**, *12*, 44.
9. Kraus, W.; Nolze, G., *J. Appl. Crystallogr.* **1996,** *29*, 301.
